# Long noncoding RNA DANCR regulates proliferation and migration by epigenetically silencing FBP1 in tumorigenesis of cholangiocarcinoma

**DOI:** 10.1038/s41419-019-1810-z

**Published:** 2019-08-05

**Authors:** Ni Wang, Chongguo Zhang, Wulin Wang, Jie Liu, Yang Yu, You Li, Mingjiong Zhang, Xianxiu Ge, Quanpeng Li, Lin Miao

**Affiliations:** 1 Medical Center for Digestive Diseases, Second Affiliated Hospital, Nanjing Medical University, Nanjing, Jiangsu Province People’s Republic of China; 2 Department of Oncology, Second Affiliated Hospital, Nanjing Medical University, Nanjing, Jiangsu Province People’s Republic of China; 3 Department of General Surgery, Second Affiliated Hospital, Nanjing Medical University, Nanjing, Jiangsu Province People’s Republic of China; 4 The Reproduction Center of Xuzhou Maternity and Child Health Care Hospital, Xuzhou, Jiangsu Province People’s Republic of China; 5 Department of Orthopaedics, the First Affiliated Hospital, Nanjing Medical University, Nanjing, Jiangsu Province People’s Republic of China; 6 Jiangsu Provincial Key Laboratory of Geriatrics, Department of Geriatrics, the First Affiliated Hospital, Nanjing Medical University, Nanjing, Jiangsu Province People’s Republic of China

**Keywords:** Oncogenes, Cell growth

## Abstract

Recently, long noncoding RNAs (lncRNAs) have been shown to play significant regulatory roles in human tumorigenesis. However, the biological function of lncRNAs in cholangiocarcinoma (CCA) remains largely unknown. In this study, DANCR was shown to be significantly upregulated in CCA. DANCR regulated the proliferation and migration of CCA cells in vitro. Moreover, downregulation of DANCR suppressed CCA cells proliferation in vivo. RNA-seq revealed that DANCR knockdown preferentially affected genes linked with cell proliferation and cell differentiation. Furthermore, mechanistic investigation validated that DANCR could bind EZH2 and modulate the histone methylation of promoter of FBP1, thereby regulating CCA cells growth and migration. Taken together, these results demonstrated the significant roles of DANCR in CCA and may provide a theoretical basis for clinical diagnosis and treatment of CCA.

## Introduction

Cholangiocarcinoma (CCA), an extremely malignant tumor that arises from cholangiocytes, has been a major health burden worldwide for decades^1^. Given the lack of sensitive indicators, the diagnosis of the majority of CCA cases typically occurs at a late stage, resulting that patients with unresectable tumors only have a median overall survival <12 months^2,3^. Therefore, it is imperative to identify novel diagnostic and therapeutic targets by deciphering the carcinogenesis and progression mechanisms underlying CCA to improve patient survival times.

Long noncoding RNAs (lncRNAs), the RNA transcripts longer than 200 nucleotides with little or no protein-coding potential^4,5^, have been shown to exhibit critical function in tumorigenesis, including CCA^6,7^. In addition, as a regulator in the process of epigenetics, lncRNAs could regulate gene expression in chromatin modification, transcription and posttranscription process^8^. For instance, lncRNA HOXC-AS3 promotes GC cell proliferation and migration through modifying the transcription of target genes by an interaction with YBX1^9^. Overexpressed UCA1 could regulate migration and invasion potential of CCA cells through activating AKT/GSK-3β axis to upregulate CCND1 expression^10^.

Long noncoding RNA DANCR (Differentiation antagonizing nonprotein coding RNA), also known as ANCR or SNHG13, is an 855-nucleotide lncRNA located at human chromosome 4q12 and was first identified as a suppressor during epidermal progenitor cell differentiation^11^. Recently, DANCR has been found to be aberrantly expressed and play important roles in a variety of tumors^12–20^. However, the expression pattern and the exact role of DANCR in human CCA remain unclear.

In our present study, we first identified that DANCR was highly expressed in CCA tissues in comparison with normal adjacent controls. In addition, we found that DANCR could regulate cell proliferation and migration in vitro. Moreover, downregulation of DANCR suppressed CCA cell proliferation in vivo. RNA-seq analysis indicated the preference of DANCR to regulate the expression of genes related to proliferation and migration. Mechanistic investigations elucidated that DANCR could directly bind to EZH2 and then mediate the H3K27 trimethylation in promoter region of Fructose-1, 6-biphosphatase (FBP1) to inhibit the expression of FBP1, thus facilitating CCA tumorigenesis.

## Materials and methods

### Tissue gathering and ethics statement

This study analyzed 17 CCA patients undergoing surgical treatment in the Second Affiliated Hospital of Nanjing Medical University. All specimens were immediately frozen with preservative liquid and stored in liquid nitrogen until RNA extraction. The Research Ethics Committee of Nanjing Medical University (Nanjing, Jiangsu, PR China) approved our study. Written informed consent was obtained from all patients.

### RNA extraction and qRT-PCR analysis

Total RNA was extracted from tissues or cultured cells using TRIzol reagents (Invitrogen, Carlsbad, CA, USA) according to the manufacturer’s instructions. RNA (1 µg) was then reversely transcribed into cDNA through a Reverse Transcription Kit (Takara, Dalian, China). SYBR Green (Takara, Dalian China) was used for real-time PCR analysis. The results were normalized to the expression of glyceraldehyde-3-phosphate dehydrogenase (GAPDH). The primer sequences are listed in Supplementary Table S1.

### Cell culture

CCA cell lines HuCCT1 and RBE were received from the Institute of Biochemistry and Cell Biology of the Chinese Academy of Sciences (Shanghai, China). Cell lines were cultured in DMEM (Life Technologies, Grand Island, NY, USA), which contained 10% fetal bovine serum (FBS) (ScienCell, Carlsbad, CA, USA), 100 mg/mL streptomycin, and 100 U/mL penicillin (Invitrogen, Shanghai, China), in air with 5% CO_2_ and humidity at 37 °C.

### Cell transfection

CCA cells were transfected with particular siRNAs using lipofectamine2000 (Invitrogen, CA, USA) based on the manufacturer’s instructions. Cells were harvested for analyses 48 h after transfections. Scrambled negative control siRNA (si-NC) was purchased from Invitrogen (Invitrogen, CA, USA). DANCR and EZH2 siRNAs were purchased from Realgene Biotechnology (Nanjing, China). The sh-DANCR was cloned into pENTR/U6 vector. The plasmid was transfected into CCA cells using X-tremeGEN HP DNA Transfection Reagent (Roche, Basel, Switzerland) according to the producer’s protocol.

### Cell proliferation analysis

Cell viability was determined using the CCK-8 Kit (Houston TX, USA) according to the manufacturer’s protocol. In the colony formation test, transfected cells were placed in six-well plates with medium containing 10% FBS. After 14 days, colonies were fixed with methanol and stained with 0.1% crystal violet (Sigma). The stained colonies were counted. Edu assays were performed using the Edu Cell Proliferation Assay Kit (Ribobio, Guangzhou, China) following the manufacturer’s instructions. Then, the percentages of Edu-positive cells were examined in the sample. All experiments were performed in biological triplicates.

### Cell migration assays

For migration assays, 3 × 10^4^ transfected cells in media with 1% FBS were added to the upper insertion chamber (Millipore, Billerica, MA, USA), while the lower chamber contained the medium with 10% FBS. After incubation for 24 h, the remaining cells on the upper membrane were removed with a cotton swab. Cells migrating through the membrane were dyed with methanol, stained with 0.1% crystal violet, and imaged with an IX71 inverted microscope (Olympus, Tokyo, Japan). All wells were assessed thrice.

### Western blot assay and antibodies

Cells protein lysates assessed by 10% sodium dodecyl sulfate-polyacrylamide gel electrophoresis were transferred to nitrocellulose membranes (Sigma-Aldrich, St Louis, MO) for incubation with specific antibodies. ECL chromogenic substrates were measured by a density meter (Quantity One software; Bio-Rad). GAPDH antibody was used as the control. Anti-EZH2 was purchased from Proteintech (Wuhan, China), and anti-FBP1 was purchased from Abcam (Cambridge, UK).

### Flow cytometric analysis

After 48 h of transfection with si-NC or si-DANCR, HuCCT1 and RBE cells were harvested by trypsinization. After double staining with fluorescein isothiocyanate (FITC)-Annexin V and propidium iodide (BD Biosciences, Franklin Lakes, NJ, USA), the cells were analyzed by flow cytometry (FACScan; BD Biosciences).

### In vivo tumor formation assays

Four-week-old athymic male mice purchased from the Animal Center of Nanjing University (Nanjing, China) were maintained under certain pathogen-free conditions. HuCCT1 cells stably transfected with sh-DANCR, DANCR or FBP1 overexpression vector, or empty vector were harvested and washed with phosphate-buffered saline. Cells resuspended at 2 × 10^7^ cells/mL were xenografted into the ventral side of each BALB/c male nude mice. Then, every 2 days, tumor volumes were calculated using the formula: *V* = 0.5 × *D* × *d*^2^ (*V*, volume; *D*, longitudinal diameter; and *d*, latitudinal diameter). On the 18th day after injection, the mice were asphyxiated in CO_2_ and the tumor weights were determined and analyzed. This study was in strict compliance with the Guide for the Care and Use of Laboratory Animals of the National Institutes of Health. Committee on the Ethics of Animal Experiments of Nanjing Medical University licensed the protocol.

### Chromatin immunoprecipitation (ChIP) assays

ChIP assays were conducted with EZ-CHIP Kit in accordance with the manufacturer’s description (Millipore, USA). The antibodies for EZH2 and H3 trimethyl Lys 27 (trimethylation of lysine residues 27 of histone 3 (H3K27me3)) were bought from Millipore and Abcam, respectively. The ChIP primer sequences are listed in Supplementary Table S1. Quantification of immunoprecipitated DNA was performed using qPCR. ChIP data were calculated as percentages relative to the input DNA from the following equation: 2^[Input Ct−Target Ct]^ × 0.1 × 100.

### RNA immunoprecipitation (RIP) assays

RIP assays were conducted using a Magna RIP™ RNA-Binding Protein Immunoprecipitation Kit (Millipore, Billerica, MA, USA) in accordance with the manufacturer’s protocols. The EZH2 antibody for RIP assays was obtained from Millipore (Billerica, MA, USA).

### In vitro transcription assays and RNA pull-down assays

In vitro translation assays were performed using mMESSAGE mMACHINE™ T7 Transcription Kit following the manufacturer’s instruction (Invitrogen, CA, USA). Then DANCR RNAs were labeled by desthiobiotinylation by Pierce RNA 3′ End Desthiobiotinylation Kit (Magnetic RNA-Protein Pull-Down Kit, Components, Thermo). RNA pull-down assays were performed through Pierce Magnetic RNA-Protein Pull-Down Kit according to the manufacturer’s instruction (Magnetic RNA-Protein Pull-Down Kit, Thermo).

### Whole transcriptome deep sequencing

Total RNA from DANCR knockdown and control HuCCT1 cells were isolated and quantified. The concentration of each sample was measured with NanoDrop 2000 (Thermo Scientific, USA). Quality evaluation was conducted with Agilent2200 (Agilent, USA). The sequencing library of each RNA samples was prepared using Ion Proton Total RNA-Seq Kit v2 (Life technologies, USA). See Supplementary Tables S2 and S3 for the data of the six samples.

### Statistical analysis

GraphPad Prism5 (GraphPad Software, La Jolla, USA) was used for statistical analysis. The significance of the differences between groups was assessed with Student’s *t*-test, #2 test or Wilcoxon test, as appropriate. The dysregulated genes from GEO datasets were obtained by limma R package and edger R package, respectively. All resulting data were recounted as the mean ± SD. A two-sided *P*-value < 0.05 was considered statistically significant.

## Result

### DANCR is upregulated in human CCA tissues

**Fig. 1:**
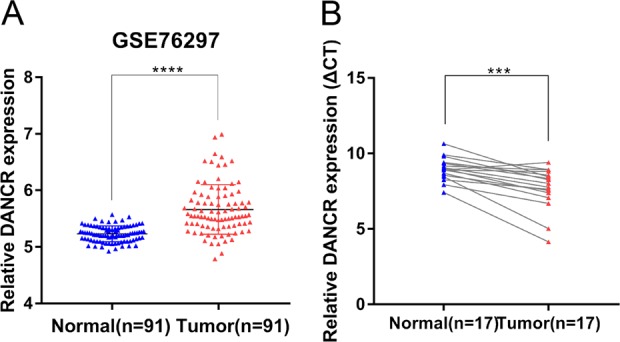
DANCR is overexpressed in CCA tissues. **a** DANCR is overexpressed in GEO datasets (GSE76297). **b** DANCR was measured in 17 pairs of CCA tissues by qRT-PCR. DANCR level in CCA tissues was higher than those in nontumorous tissues. All experiments were performed in biological triplicates. Error bars indicate means ± SD. ***P < 0.001; ****P < 0.0001

To obtain the differential expression of lncRNAs in CCA, we performed an integrative analysis of the raw microarray data downloaded from GEO database (GSE76297; including 91 pairs of tumor tissue samples and normal tissue samples, and one unmatched normal tissue sample; we abandoned the data of the single normal tissue specimen and conducted paired *t*-test on 91 pairs of tissue samples data)^21^ and focused on the lncRNA DANCR which is highly expressed in human CCA tissues (Fig. 1a). We further verified the remarkable increased expression levels of DANCR in a cohort of 17 paired CCA tumor tissues and adjacent nontumor tissues, which was consistent with the results from high-throughput data (Fig. 1b). These findings prompted us to explore the possible carcinogenic role of DANCR in CCA growth.

### DANCR regulates CCA cell proliferation and migration in vitro

**Fig. 2:**
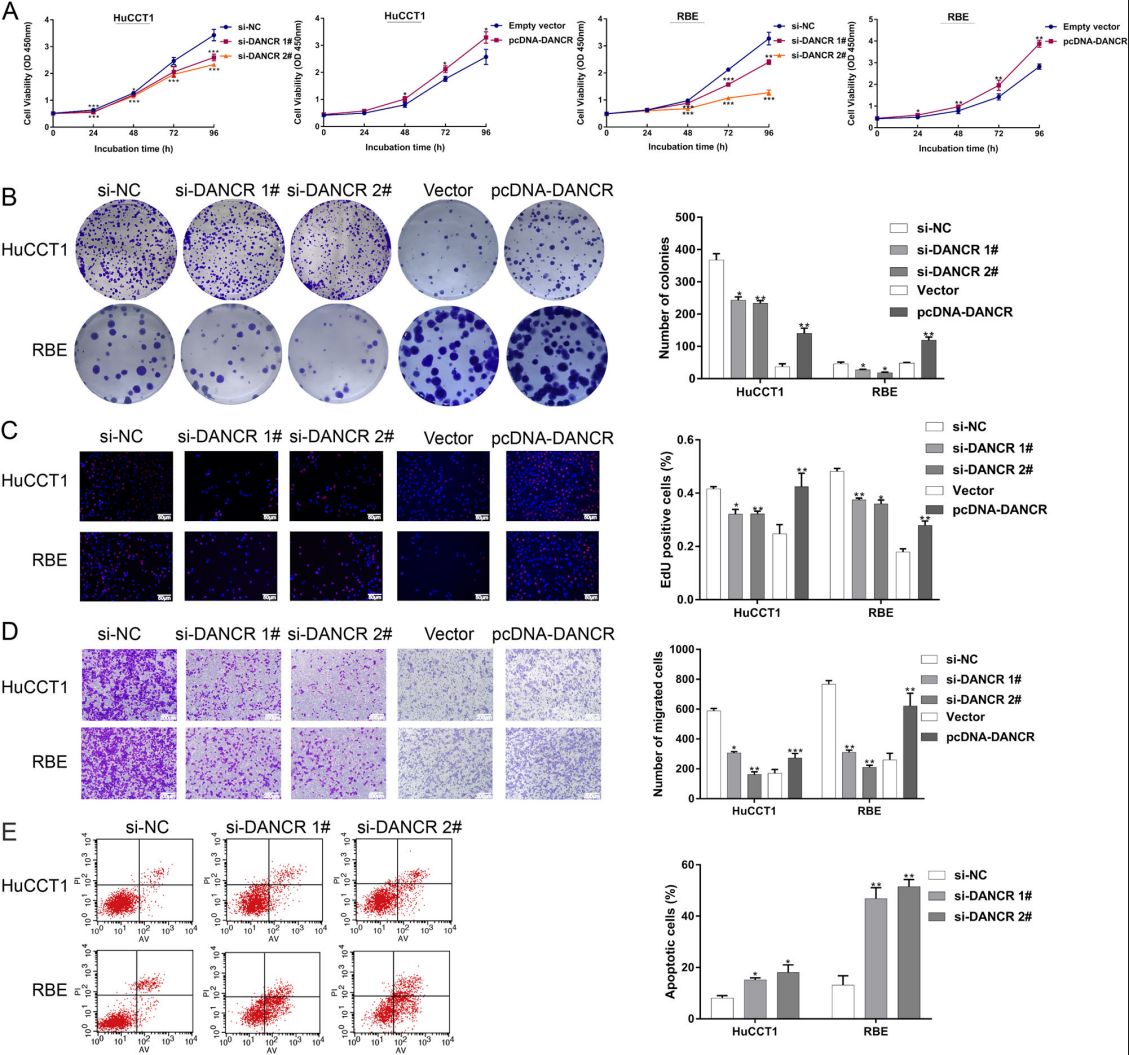
DANCR regulates CCA cell proliferation and migration in vitro. **a** CCK-8 assays were used to detect the viability of HuCCT1 and RBE cells after transfection of overexpression plasmid and knockdown of DANCR. **b** Colony formation assays of si-DANCR-treated or pcDNA3.1-DANCR-treated CCA cells. Colonies were counted and captured. **c** Proliferating CCA cells were marked with EdU (red). Cell nuclei were stained with DAPI (blue). **d** The migration of CCA cells after transfection was investigated using transwell assays. **e** At 48 h after transfection, the apoptotic rates of CCA cells were measured by flow cytometry. LL dead cells, UL viable cells, LR early apoptotic cells, UR terminal apoptotic cells. All experiments were performed in biological triplicates. Error bars indicate means ± SD. *P < 0.05; **P < 0.01; ***P < 0.001. The expression of DANCR following stably transfection of HuCCT1 cells with sh-DANCR

To evaluate the biological function of DANCR in CCA, we regulated the exogenous knockdown or overexpression of DANCR by siRNA or plasmid, respectively (Supplementary Fig. S1A, B). Then, CCK-8 experiments revealed that silencing DANCR could remarkably inhibit HuCCT1 and RBE cell viability, while the cells exhibiting increased DANCR expression levels showed a higher cell viability rate than controls (Fig. 2a). Colony formation analysis demonstrated that the clonogenic survival of CCA cells was also greatly attenuated with DANCR knockdown. By contrast, overexpression of DANCR could enhance the growth ability (Fig. 2b). Similarly, EdU-staining assays further validated the significant impact of dysregulated DANCR on CCA cell proliferation (Fig. 2c). Next, using a transwell assay, we found that HuCCT1 and RBE cell migration were observably impaired after DANCR knockdown. In contrast, DANCR overexpression promoted CCA cell migration (Fig. 2d). Furthermore, we performed flow cytometry to investigate whether DANCR was involved in CCA cell apoptosis. Consistent with expected results, downregulation of DANCR improved HuCCT1 and RBE cell apoptotic rates (Fig. 2e). Taken together, these results indicated that DANCR could accelerate the proliferation and migration of CCA cells in vitro.

### Downregulation of DANCR suppresses CCA cell Tumorigenesis in vivo

**Fig. 3:**
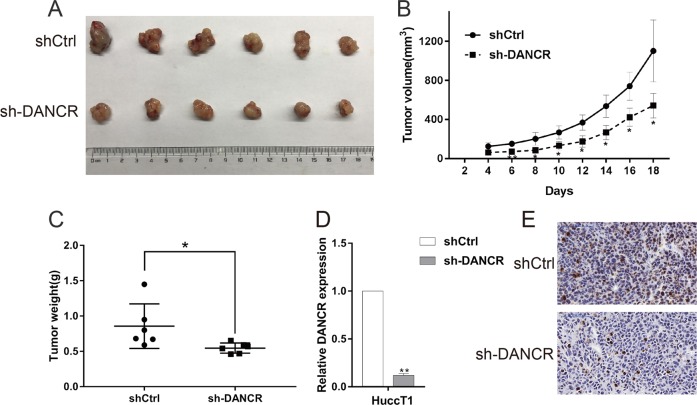
DANCR regulates CCA cell tumor growth in vivo. **a** Stable DANCR knockdown HuCCT1 cells were injected into the nude mice. **b** Tumor volumes were calculated after injection every 2 days. **c** Tumor weights are represented as the means of tumor weights ± S.D (standard deviation). **d** qRT-PCR showed that the average expression of DANCR in sh-DANCR-expressing tumor tissues was lower than that in the control group. **e** Immunohistochemistry analysis revealed that the tumors developed from sh-DANCR cells displayed lower Ki-67 staining than the control group. *P < 0.05; **P < 0.01

We further used a xenograft mouse model to verify the influence of DANCR on tumorigenesis of CCA in vivo. HuCCT1 cells stably transfected with sh-DANCR or empty vector were subcutaneously inoculated into male nude mice. Eighteen days following the injection, tumors that formed in the sh-DANCR group grew substantially slower compared with those in the control group (Fig. 3a, b). At the end of the experiment, the average weight of the tumor with sh-DANCR was markedly reduced compared with the control with empty vector (Fig. 3c). The decreased expression of DANCR was confirmed in sh-DANCR-expressing tumor tissues compared with control tumors (Fig. 3d). Correspondingly, tumors formed from stably sh-DANCR-transfected HuCCT1 cells exhibited decreased positivity for Ki-67 than tumors from the control cells (Fig. 3e). These findings indicated that knockdown of DANCR could inhibit tumor growth in vivo, which further suggested the important role of DANCR in CCA growth.

### Downstream targets of DANCR in CCA

**Fig. 4:**
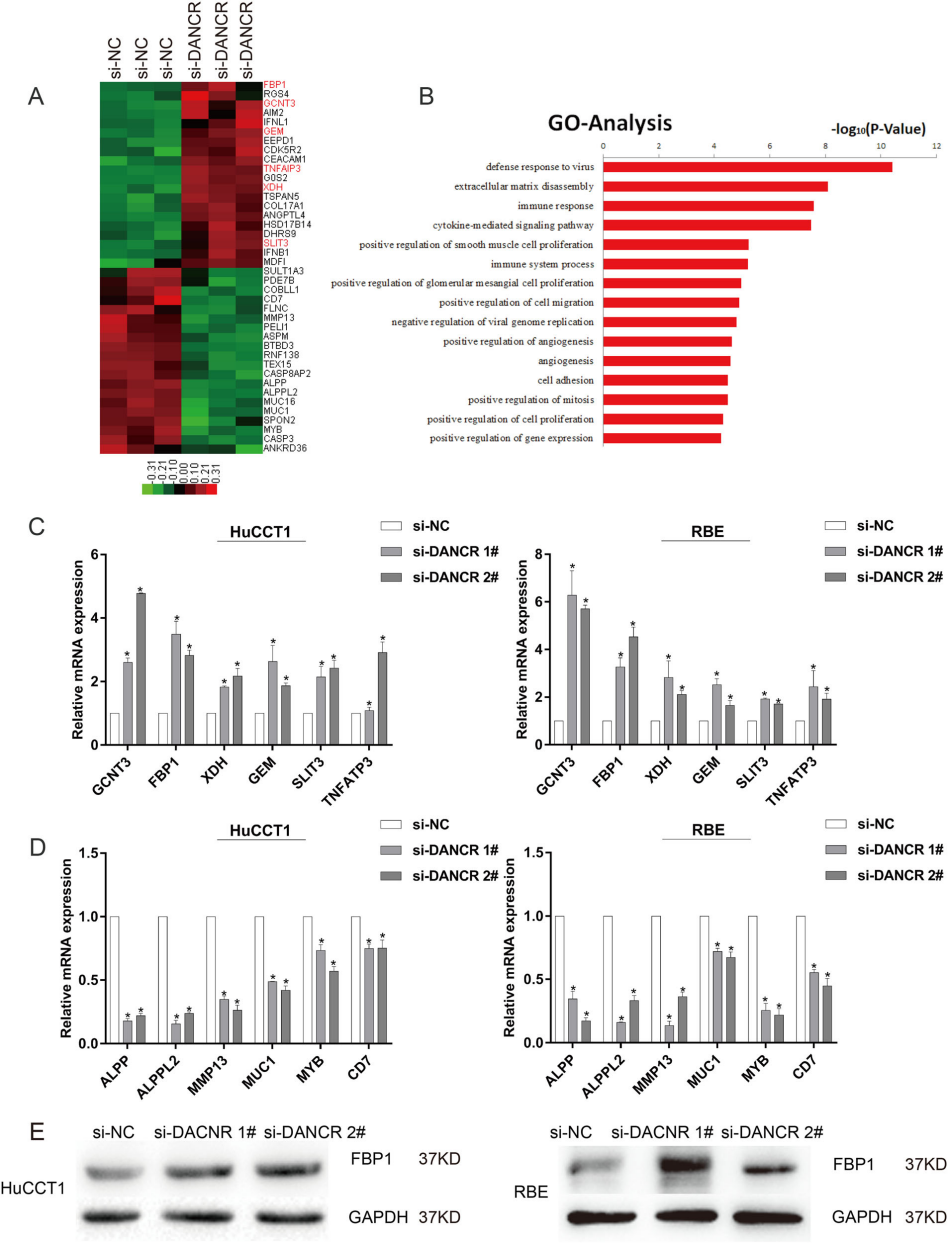
RNA-seq after DANCR knockdown in CCA cells. **a** Mean-centered, hierarchical clustering of gene transcripts altered (≥1.5-fold change) in si-NC-treated cells and siRNA DANCR-treated cells, with three replicates. **b** Gene ontology analysis for all genes with altered expression levels after knockdown of DANCR. **c**, **d** The altered mRNA levels of genes were selectively confirmed by qRT-PCR in cells with DANCR knockdown. **e** Western blot assays were used to confirm the protein levels of FBP1 after transfecting with si-DANCR. Error bars indicate means ± SD. *P < 0.05

To explore the potential target genes related to DANCR in CCA cells, we performed RNA transcriptome sequencing in control or si-DANCR. A common set of 418 mRNAs showed ≥1.5-fold significantly upregulated or downregulated abundance in HuCCT1 cells as a consequence of DANCR knockdown (Fig. 4a, Supplementary Table S2 and S3). The in-depth evaluation of the genetic ontology analysis indicated that the most obviously representative biological phenomena included pathways involved in cell proliferation and cell differentiation (Fig. 4b). Prospectively, as shown in Fig. 4a, sequencing results indicated that many proliferation and migration-associated tumor suppressor genes (e.g., GCNT3, FBP1, XDH, GEM, SLIT3, TNFATP3, et al.) or oncogenes (e.g., ALPP, ALPPL2, MMP13, MUC1, MYB, CD7, et al.) were differentially up- or down-expressed, respectively, subsequent to DANCR knockdown. The regulation of these genes was selectively verified using qRT-PCR in DANCR-silenced HuCCT1 and RBE cells (Fig. 4c, d). Noticeably, after DANCR knockdown, the significant elevation of FBP1 whose antineoplastic effect has been elucidated in CCA aroused our concern^22^. FBP1, which resides on human chromosome 9q22^23^, is a pivotal gluconeogenesis regulatory enzyme^24^. In addition to CCA, accumulating evidence has revealed the anticancer role of FBP1 in various cancer types^23–27^. Furthermore, the negative regulatory relationship between DANCR and FBP1 was confirmed on the protein level consistently, which prompted us to focus on FBP1 as a key target gene of DANCR for the following mechanism research (Fig. 4e).

### DANCR silences FBP1 epigenetically through binding to EZH2

**Fig. 5:**
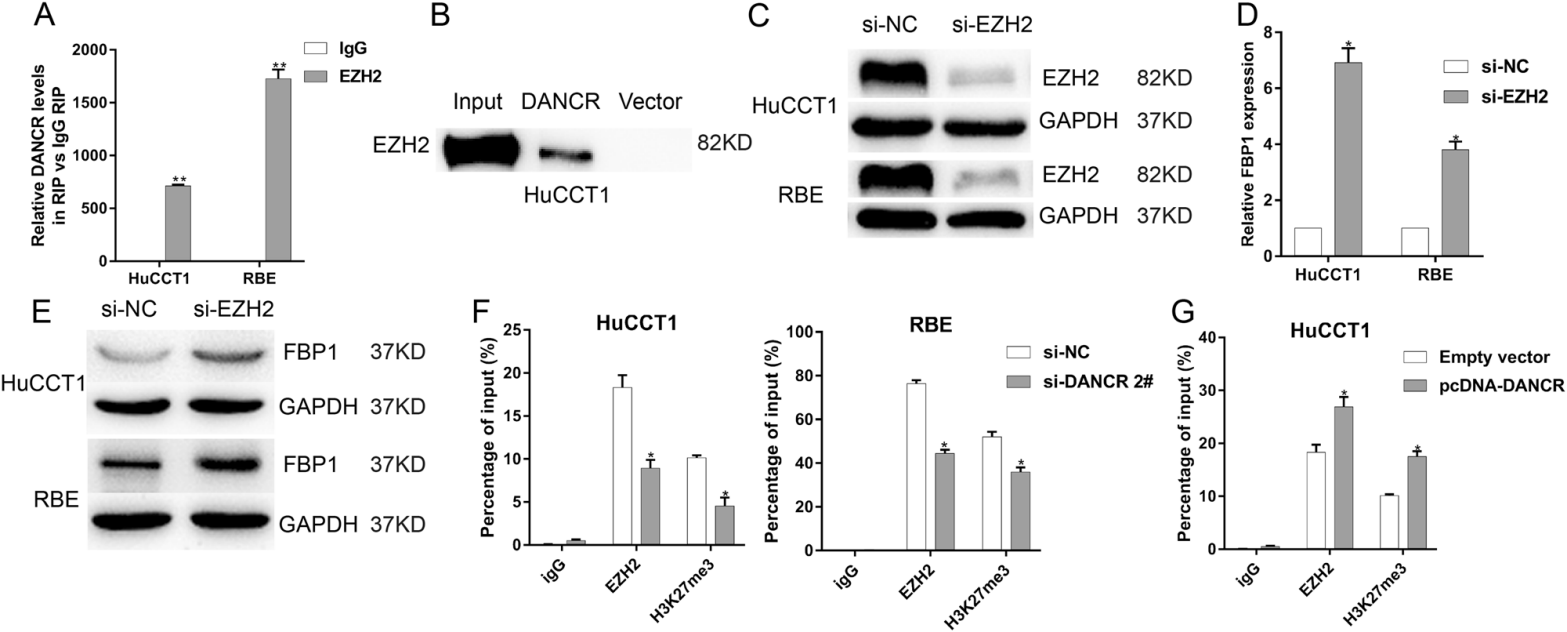
DANCR binds to EZH2 to suppress FBP1 epigenetically. **a** RIP experiments established that DANCR could interact with EZH2. **b** In vitro transcription, pull-down assays showed that desthiobiotinylation-DANCR could bind EZH2 in HuCCT1 cells. **c** Western blot assays detected the altered protein level of EZH2 following treatment of CCA cells with si-EZH2. **d**, **e** The mRNA and protein levels of FBP1 expression after knockdown of EZH2 were detected by qPCR and western blot, respectively. **f** ChIP assays identified the enrichment of EZH2 and H3K27me3 in the promoter region of FBP1, and this enrichment was decreased after silencing DANCR in HuCCT1 and RBE cell line. **g** The enrichment of EZH2 and H3K27me3 was increased after DANCR overexpression in HuCCT1. Enrichment was quantified relative to input controls. Antibody directed against immunoglobulin G (IgG) was used as a negative control. Error bars indicate means ± SD. *P < 0.05; **P < 0.01

Recent studies have uncovered that a significant number of lncRNAs could bind to chromatin modifying enzymes and thus have synergistic effects in epigenetic activation or silencing of target gene expression^28^. Remarkably, it is reported that DANCR could bind to enhancer of zest homolog 2 (EZH2) to regulate downstream genes at the epigenetic level in prostate cancer and gastric cancer^29,30^. EZH2, the critical element of a methyltransferase named polycomb repressive complex 2 (PRC2)^31^, could serve as a master regulator of transcription that contributes to cancer development and progression via promoter hypermethylation of target gene^32^. Interestingly, transcriptional inactivation of FBP1 expression due to promoter region DNA hypermethylation was observed in hepatocellular carcinoma^25^, colon cancer^33^, gastric carcinogenesis^34^, basal-like breast cancer^24^ and lung cancer^35,36^. Furthermore, recent studies have reported that epigenetic modifications like DNA and histone methylation could exhibit functional cooperation in heritable repression of gene activity^37–40^. Therefore, we hypothesized that DANCR might modulate the expression of FBP1 through binding to EZH2. As shown in Fig. 5a, compared with nonspecific IgG control fraction, sufficient precipitate of endogenous DANCR was observed in the anti-EZH2 antibodies fraction, which suggested the interaction between EZH2 and DANCR. Then, RNA pull-down assays demonstrated that DANCR RNA, but not vector, specially retrieved EZH2 from HuCCT1 nuclear extract (Fig. 5b), which further verified the bond of EZH2 and DANCR. To investigate whether EZH2 could negatively regulate transcription of FBP1 via H3K27me3, the following experiments were further conducted. After the decreased expression of EZH2 using effective siRNAs in HuCCT1 and RBE cell lines, the mRNA and protein levels of FBP1 increased potently (Fig. 5c–e). Given that EZH2 regulates target gene transcription through promoter binding and results in histone modification via H3K27me3 in this region, we examined whether loss or gain of DANCR affected enrichment of EZH2 and H3K27me3 in the FBP1 promoter region. Subsequently, ChIP assays followed by qPCR demonstrated that DANCR knockdown quelled EZH2 binding and H3K27me3 levels in the FBP1 promoter (Fig. 5f). Conversely, the binding to EZH2 and the level of H3K27me3 were elevated through the promoter of FBP1 when DANCR was overexpressed in HuCCT1 (Fig. 5g). The above results confirmed that DANCR could repress FBP1 expression epigenetically partly by binding with EZH2 to catalyze H3K27me3 in the FBP1 promoter region, promoting CCA cell growth and migration.

### FBP1, the bona target of DANCR, is a tumorigenic suppressor in CCA cells

**Fig. 6:**
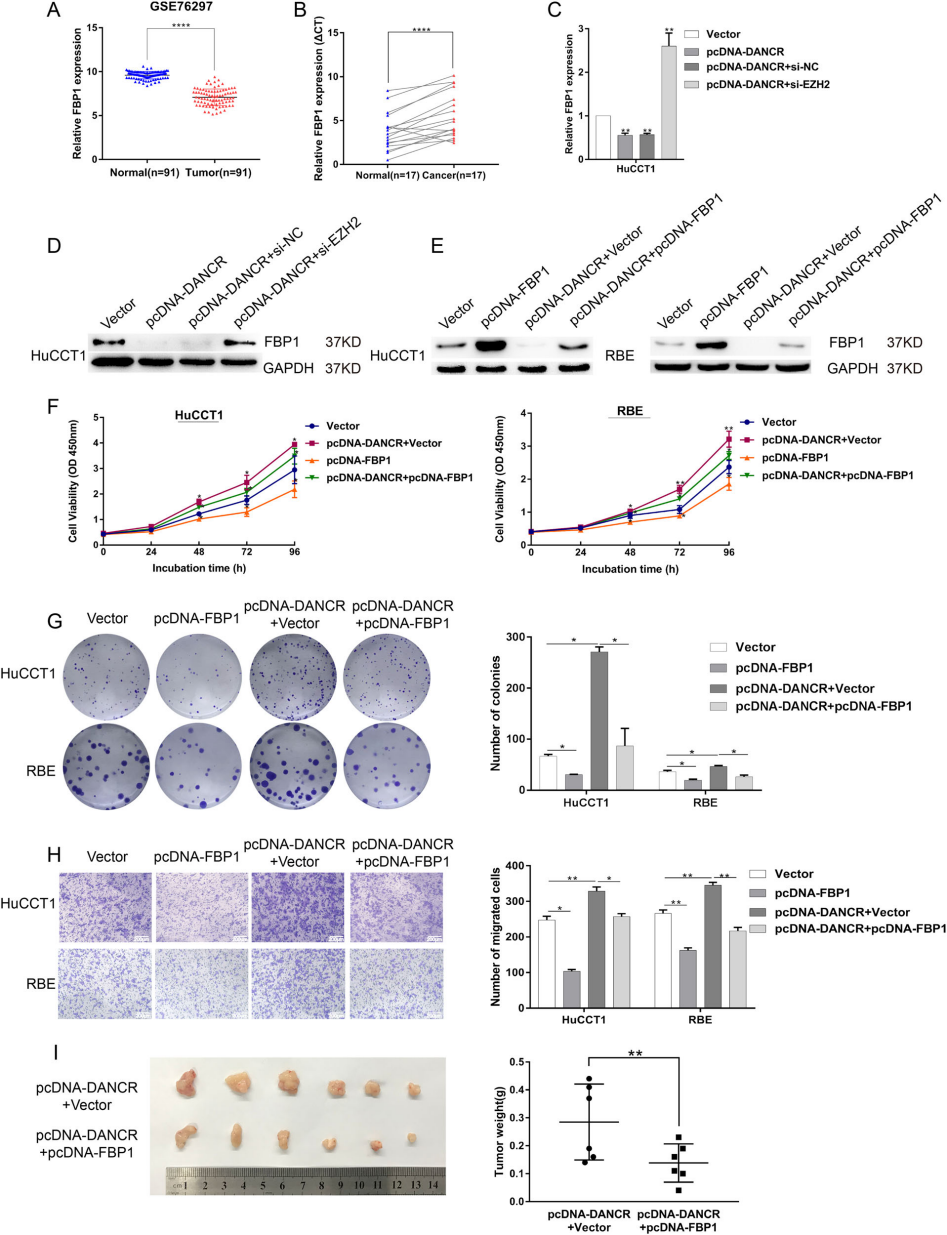
FBP1 suppresses CCA cell proliferation and metastasis and counterbalances DANCR activity. **a** GES76297 data uncovered the low expression levels of FBP1 in CCA. **b** Relative expression of FBP1 was detected in 17 pairs of CCA tissues by qRT-PCR. **c, d** The mRNA and protein level of FBP1 was confirmed by qRT-PCR and western blot in DANCR-overexpressing HuCCT1 cells and cells simultaneously transfected with si-EZH2. **e** Western blot assays detected the altered protein level of FBP1 in HuCCT1 and RBE cells transfected with vector/pcDNA-FBP1/pcDNA-DANCR or cotransfected with pcDNA-DANCR and pcDNA-FBP1. **f–h** These cells were analyzed by CCK-8 assays (**f**), colony formation (**g**), and transwell assays (**h**). **i** HuCCT1 cells stably transfected with DANCR overexpression vector and cotransfected with DANCR and FBP1 overexpression vector were injected into the nude mice. Tumor weights were measured after tumor removal. Error bars indicate means ± SD. *P < 0.05; **P < 0.01

To investigate the functional phenotype of FBP1 in CCA cells, we assessed the expression of FBP1 using the GSE76297 dataset and found reduced FBP1 expression in cancer tissues compared with control normal tissues (Fig. 6a). Consistently, the downregulation of FBP1 in CCA was confirmed by qRT-PCR in 17 pairs of CCA tissues (Fig. 6b). The negative regulatory relationship between DANCR and FBP1 was confirmed after the overexpression of DANCR in HuCCT1 (Fig. 6c, d). Furthermore, the inhibition of FBP1 by DANCR was reversed following transcription with si-EZH2 on both the mRNA and protein levels (Fig. 6c, d). Then, we performed western blot assays to detect the protein level of FBP1 in HuCCT1 and RBE cells transfected with vector/pcDNA-FBP1/pcDNA-DANCR or cotransfected with pcDNA-DANCR and pcDNA-FBP1 (Fig. 6e). Moreover, we found that elevated FBP1 evidently impaired proliferation and migration ability of CCA cell lines. Furthermore, overexpression of FBP1 was also capable of partially reversing DANCR-mediated growth and migration promotion (Fig. 6f–h). We injected DANCR-overexpressing HuCCT1 cells, or the HuCCT1 cells stably cotransfected with DANCR and FBP1 overexpression vector into nude mice. Correspondingly, FBP1 could partially rescue DANCR-induced cell proliferation in vivo (Fig. 6i). Taken together, our study demonstrated that DANCR might combine with EZH2 and inhibited FBP1 expression epigenetically, thus promoting CCA malignancy.

## Discussion

To date, a large proportion of the research on cancers was concentrated on oncogenes and tumor suppressors with protein-coding potential that may be effectively used as clinical biomarkers. However, accumulating evidence has revealed that <2% of the human genome is subsequently translated and most genomes produce noncoding RNA (ncRNA) transcriptions^41–43^. Moreover, the irreplaceable position of lncRNA was emphasized in a variety of cancers, including CCA. For example, our previous studies revealed that PVT1 could function as an oncogenic lncRNA in human CCA^44^. Lu et al. found that lncRNA AFAP1-AS1 was upregulated in CCA and could regulate cell migration and invasion with downregulation of MMP-2 and MMP-9^6^. These suggested that lncRNA may play an important role in CCA. In this study, utilizing publicly available lncRNA raw microarray data of CCA, we identified the overexpression of DANCR in CCA. DANCR, which was first identified as a suppressor in the progression of epidermal cell differentiation^11^, was shown to be positively associated with tumors progression and poor prognosis in many different types of cancers^12–20^. Nevertheless, a few studies also revealed the opposite function of DANCR as a tumor suppressor in breast cancer, renal cell carcinoma and nonsmall cell lung cancer^20,45–47^, which may result from the obvious tissue-specific expression patterns of lncRNAs than protein-coding genes^48,49^. These results suggested the tissue specificity of DANCR expression. However, the possible role of DANCR remains undocumented in CCA, which further arouse our interest to explore.

As the results indicated in our study, inhibition of DANCR could repress the proliferation and migration of CCA cells in vitro. Moreover, DANCR downregulation suppressed CCA proliferation in vivo. Although we have identified the carcinogenic property of DANCR in CCA, DANCR-related regulatory mechanism in CCA remains unclear. Furthermore, we conducted RNA transcriptome sequencing after knockdown of DANCR. Moreover, gene ontology analysis suggested that gene expression profiles were mainly related to proliferation and differentiation.

It is reported that a significant number of lncRNAs have been shown to function in cooperation with chromatin modifying enzymes to activate or silence target gene expression epigenetically^28^. Our mechanistic result data revealed that DANCR could bind to EZH2, the critical element of a methyltransferase named PRC2^31^, suggesting that DANCR may promote CCA progress via transcriptionally regulating target genes that are linked to cell proliferation and cell migration through interaction with EZH2. Increasing studies have proposed that EZH2 gene played an oncogenic role in cell proliferation, differentiation, invasion, and metastasis in various human malignant tumors^32,50,51^. Furthermore, EZH2 could interact with lncRNAs to catalyze the H3K27me3 in the promoter regions of the target gene, thereby mediating transcriptional silencing^52,53^. For example, lincRNA HOTAIR was identified to promote breast metastasis by binding to EZH2 and transcriptionally repressing HOX loci^54^. Our previous study found that lncRNA SNHG1 could act as an “oncogene” for CCA partly via suppressing the expression of CDKN1A by binding with EZH2^40^. In our present study, we found that DANCR could bind to EZH2, thus transcriptionally regulating target proliferation and migration-related genes, thus promoting CCA progress.

Among these DANCR-regulated target genes, FBP1, a tumor suppressor gene identified in various cancers aroused our concern. FBP1 was significantly negative correlated with DANCR mRNA and protein levels in CCA. The FBP1 gene encodes a rate-limiting gluconeogenic enzyme. Loss of the FBP1 gene has been reported to accelerate tumor progression by enhancing aerobic glycolysis, thereby resulting in poor prognosis in clear cell renal cell carcinoma^23^ and breast cancer^24^. Besides renal and breast cancer, the anticancer role of FBP1 has been verified in various human cancer types^23–27^, including CCA^22^. Here, we provided evidence for low expression of FBP1 in CCA, and overexpression of FBP1 inhibited the proliferation and migration of CCA cells. FBP1 transcriptional inactivation resulting from DNA methylation of the FBP1 promoter region has been observed in various cancers^23–27^. We found that histone methylation (H3K27me3) mediated by DANCR could contribute to the lower expression of FBP1 in CCA as well. Furthermore, studies have showed that histone methylation and DNA methylation typically have the cooperative function in the inactive expression of target genes heritably^37,38,55^. As discovered by our study, transcriptional downregulation of FBP1 could be partly mediated by the DANCR-EZH2 complex through the promoter H3K27me3, thus facilitating CCA cell proliferation and migration.

In summary, our study showed that upregulated DANCR could promote CCA progress through transcriptional inactivation of the target tumor suppressor gene FBP1 epigenetically, which revealed that DANCR could provide a theoretical basis for clinical diagnosis and treatment of CCA.

## Supplementary information

Supplementary Figure S1

Supplementary Figure S1 Legent

Supplementary table S1

Supplementary table S2

Supplementary table S3
