# Supplementary figures and images for: Long noncoding RNA DANCR regulates proliferation and migration by epigenetically silencing FBP1 in tumorigenesis of cholangiocarcinoma
Source: Cell Death Dis. 2019 Aug 5;10(8):585. doi: 10.1038/s41419-019-1810-z (PMC6683119; doi:10.1038/s41419-019-1810-z)

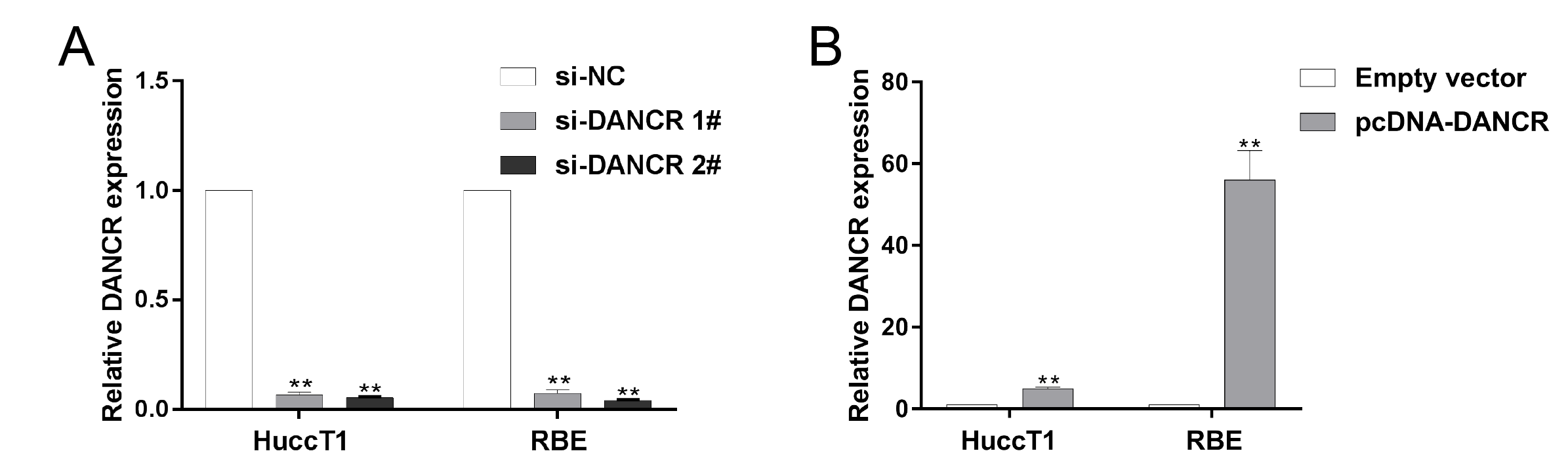

Supplement: Supplementary file 1 — Supplementary Figure S1 [file 41419_2019_1810_MOESM1_ESM.tif]
