# Supplementary material for: Long noncoding RNA DANCR regulates proliferation and migration by epigenetically silencing FBP1 in tumorigenesis of cholangiocarcinoma
Source: Cell Death Dis. 2019 Aug 5;10(8):585. doi: 10.1038/s41419-019-1810-z (PMC6683119; doi:10.1038/s41419-019-1810-z)
Supplement: Supplementary file 2 — Supplementary Figure S1 Legent [file 41419_2019_1810_MOESM2_ESM.docx]

Figure. S1 (A) The expression of DANCR following treatment of HuCCT1/RBE cells with siRNAs. (B) The expression of DANCR following transfection of HuCCT1/RBE cells with pcDNA3.1+ DANCR.
